# Perceptions of essential oral health care and locus of control among adults in a developing country

**DOI:** 10.1016/j.jobcr.2026.101487

**Published:** 2026-07-08

**Authors:** Aparna K.S., Ritu Duggal, Vijay P. Mathur, Harsh Priya, Bharathi M. Purohit, Sarah Paul, Neha Chauhan

**Affiliations:** Centre for Dental Education and Research (CDER), All India Institute of Medical Sciences (AIIMS) New Delhi, India

**Keywords:** Essential oral health care, Locus of control, Perception, Barriers, India

## Abstract

**Background:**

Oral diseases are among the most prevalent global health problems, disproportionately affecting disadvantaged populations. Essential Oral Health Care (EOHC), endorsed by the World Health Organization, comprises basic promotive, preventive, and curative services that should be universally accessible. Public perceptions and psychological factors, particularly Locus of Control (LOC), strongly influence oral health behaviors and service use. This study assessed adults’ perceptions of Essential Oral Health Care (EOHC) services and examined the association of Locus of Control (LOC) with sociodemographic factors.

**Methods:**

A cross-sectional study was conducted among 150 adults residing in Trilokpuri, New Delhi. Data were collected through interviewer-administered questionnaires on six domains: perceived needs, essential services, service adequacy, barriers to utilization, self-rated oral health and LOC. Statistical tests, including Mann- Whitney *U* test, Spearman correlation test, and multivariable logistic regression, were applied.

**Results:**

Most participants (68.0%) recognized oral health as part of general health, yet only 34.0% reported a dental visit in the past year. Pain relief (42.0%) was the most commonly perceived essential service. Cost and waiting time were major barriers. Internal LOC was observed in 38.7% of participants and was positively associated with higher socioeconomic status (SES) (p < 0.05).

**Conclusion:**

Adults demonstrated limited awareness and narrow perceptions of EOHC, with substantial access and affordability barriers. Internal LOC was significantly associated with higher SES, underscoring the need for community-based oral health education and systemic strengthening of affordable EOHC services.

## 1 Background

Oral diseases constitute a major public health burden globally, affecting individuals across all age groups.^1^ These conditions—most notably dental caries, periodontal diseases, edentulism, and oral cancers—are among the most prevalent non-communicable diseases (NCDs) and have significant implications for general health, quality of life, and economic productivity.^2^ The socioeconomically weaker and geographically disadvantaged communities in India, such as rural and tribal areas, often bear an unfair burden of oral diseases.^3^ Despite being largely preventable and treatable when addressed early, oral conditions often remain untreated in India. These conditions can leave patients in pain, susceptible to infections, lose teeth, and affect systemic health. Moreover, they have a greater impact on quality of life and social participation.^4^^,^^5^

Essential Oral Health Care (EOHC) refers to the minimum interventions required to maintain healthy teeth, healthy gums and healthy other oral tissues. It consists of promotive, preventive and minimally invasive curative services.^6^^,^^7^ WHO recognizes EOHC as an important element of UHC and a pathway to the SDGs.^6^^,^^8^ The WHO's Basic Package of Oral Care (BPOC), comprising Oral Urgent Treatment (OUT), Affordable Fluoride Toothpaste (AFT), and Atraumatic Restorative Treatment (ART), has been advocated to serve as the minimum standard of affordable and accessible care globally.^9^^,^^10^ In India, EOHC aligns conceptually with primary health care delivery, where prevention, early identification, referral, and basic essential services are intended to be provided through the public sector platform, including Primary Health Centres and Health and Wellness Centres, with linkage to higher facilities for definitive care. Despite this alignment, evidence suggests that the reach and uptake of such necessary services are quite limited. National oral health surveys and service statistics are principally concerned with clinical prevalence. However, individual perspectives such as perceived needs, expectation of care and barriers to access services are not as extensively measured.^4^^,^^9^

Studies across populations consistently describe multi-level barriers that shape dental care seeking, including limited awareness, misperceptions about the need or safety of dental care, cost, accessibility constraints, and health-system factors 11, 12, 13, 14. Importantly, these barriers do not operate only at the level of service availability; they also reflect how individuals perceive control over health and the value of preventive action. Locus of Control (LOC) is a health psychology construct describing whether individuals attribute health outcomes primarily to their own actions (internal LOC) or to external forces such as fate, chance, or powerful others (external LOC). Internal LOC is generally associated with preventive behaviors and better treatment adherence, whereas external LOC is often linked with delayed care seeking and lower engagement in prevention.^15^^,^^16^ Oral health research similarly indicates that internal LOC correlates with more consistent hygiene practices and preventive dental visits, while external LOC is associated with poorer oral health outcomes and episodic, problem-driven service use ^15^^,^^17^, ^18^, ^19^, ^20^, ^21^.

LOC may be especially relevant in underserved Indian communities, where financial, geographic, and service barriers can foster a sense that oral health is beyond personal control. In these settings, LOC reflects both self-care behavior and empowerment to seek primary care, complete referrals, and sustain preventive routines. Indian evidence is limited, but suggests internal LOC aligns with better oral health behaviors, while socioeconomic disadvantage may worsen the impact of external LOC 22, 23, 24. Community-level evidence linking LOC with perceptions and use of EOHC particularly among underserved adults remains sparse. Assessing EOHC perceptions alongside LOC can help in understanding how adults prioritize needs, interpret barriers, and engage with essential services in India's primary health care system, guiding interventions that combine service strengthening with empowerment-focused strategies to improve EOHC uptake under UHC.^4^^,^^9^

This study aimed to assess adults’ perceptions of Essential Oral Health Care (EOHC) services and examined the association of Locus of Control (LOC) with sociodemographic factors. Specifically, the objectives were to: (i) explore perceived needs, accessibility, and coverage of EOHC; (ii) assess knowledge, attitudes, and practices; (iii) identify barriers to utilization; and (iv) analyze the influence of sociodemographic factors on LOC.

## 2 Methods

### 2.1 Study design

A community-based cross-sectional study was conducted among adults (≥18 years) from Trilokpuri, an urban outreach area of AIIMS New Delhi, between March and June 2025. Trilokpuri, in East Delhi, is a low-urban-resource community marked by dense population, lower socioeconomic strata, and limited continuous oral health services. As part of AIIMS’ urban field practice area, its healthcare relies mainly on government primary centres and mobile outreach units. The coexistence of urban overcrowding and rural-type service scarcity makes it ideal for studying access and perception gaps in Essential Oral Health Care. The study aimed to evaluate perception of essential oral health care services and their association with locus of control among the study participants.

### 2.2 Participants and sampling

The study was carried out in compliance with the Declaration of Helsinki and received ethical approval from the Institutional Ethical Committee (Ref No.: AIIMSA4054/10.06.2025). Written informed consent was obtained from all participants prior to data collection. A total of 150 community-dwelling adults were recruited using purposive sampling from the catchment population of AIIMS Rotary Midtown Eye/Dental Hospital Trilokpuri. Purposive recruitment was adopted because a complete household sampling frame was not available for probability-based selection during the study period and feasibility constraints (time and field resources) necessitated a pragmatic approach to generate an initial estimate from the outreach population; however, this may introduce selection bias and limits generalizability beyond the study setting. To improve transparency and reduce convenience-driven enrolment, field recruitment followed a standardized workflow: the outreach area was mapped and operationally segmented by lanes/blocks using local landmarks; a daily route plan ensured coverage across segments; within each segment, households were approached sequentially along one side of a lane and then the opposite side (serpentine approach) until the daily target was met; where feasible, locked households were revisited at different times. Within approached households, eligible adults were invited to participate; refusals and non-contacts were documented.

Eligible participants included both healthy individuals and those with self-reported oral health problems from this urban communities.

Based on the primary objective, assuming 50% of adults would have a positive perception of Essential Oral Health Care (EOHC)—a conservative estimate that maximizes variance—the sample size was calculated using the formula:

$$n=\frac{Z^{2}p\left(1-p\right)}{d^{2}}$$

where Z = 1.96 for 95% confidence, 80% power, p is the expected proportion (50%) and d = 0.08 (allowable error of ±8%). Following established public health sampling guidelines, allowable errors between 5 and 10% are acceptable depending on study objectives and feasibility.^25^ An allowable error of 8% was used instead of the conventional 5% to obtain a feasible sample size within available resources. As the study aimed to provide an initial estimate of adults’ perception of EOHC, a precision of ±8% was considered adequate, offering a practical balance between reliability and feasibility. This yielded a required sample of 150 participants**.**

The study subjects who satisfied the inclusion and exclusion criteria for the study were recruited. Community-dwelling adults ≥18 years willing to provide informed consent were included. Individuals unable to understand the survey language or consent process, persons with cognitive impairments affecting participation were excluded.

### 2.3 Research tool: questionnaire content, adaptation and local validation

Data were collected using a structured questionnaire developed from WHO oral health frameworks and existing LOC instruments. The tool covered six domains: demographic details; perceived need for oral health; perception of EOHC service scope; service adequacy, including accessibility and affordability; barriers to dental care; and LOC. Open-ended questions captured participants’ contextual experiences and suggestions regarding oral health services and utilization. The full questionnaire is provided in the Appendix.

The questionnaire was developed in English and translated into Hindi using a forward–backward translation procedure by two independent bilingual translators. Discrepancies were reconciled by consensus with the investigators and one subject expert. The tool was assessed for readability and comprehension during pilot study on a group of 20 study subjects. Necessary corrections and modifications were made.

### 2.4 Research tool: reliability and validity

The reliability analysis of the instrument was evaluated with Cronbach's alpha test for internal consistency. The tool showed good internal consistency (Cronbach's α = 0.82) and strong test–retest reliability (r = 0.86).

The content validity of the questionnaire was calculated by the item level content validity index (I-CVI), and the average of the Scale-level Content Validity Index (S-CVI)/Ave. Content validity was assessed by an expert panel of 7 reviewers (4 public health/community dentistry faculty, 1 biostatistician with scale-development experience, and 2 public health practitioners from urban low-resource service settings), each with ≥5 years of relevant experience. Experts rated each item for relevance and clarity on a 4-point scale 1, 2, 3, 4. I-CVI was calculated as the proportion rating 3/4; items with I-CVI ≥0.78 were retained, 0.70–0.77 revised, and <0.70 removed. S-CVI/Ave was computed as the mean I-CVI across items; the final tool achieved S-CVI = 0.92, with I-CVI values ranging from 0.86 to 1.00.

### 2.5 LOC scale operationalization

LOC was measured using 7 binary items, with responses coded 1 for the pre-defined internal orientation and 0 otherwise (including “Not sure” where applicable), yielding a total score of 0–7 (higher = stronger internal LOC). Internal responses were: “Yes” to oral health being important, interest in learning basic oral healthcare, dental visit in the past year, ability to afford treatment (Yes/Sometimes), receipt of oral health education, and satisfaction with oral health; the item on delaying/avoiding dental visits was reverse-coded (internal = “No”). LOC was categorized a priori as internal (≥4) versus external (<4) to reflect a majority (≥4/7) endorsement of internal-oriented responses.

### 2.6 Data collection and interview protocol

Data were collected through face-to-face interviews conducted by a single trained investigator at participants’ households and community gathering points. The investigator received standardized training by a senior public health dentistry faculty on study procedures, informed consent, and uniform questionnaire administration, including mock interviews and supervised field practice and on-site completeness checks to ensure data quality. A standardized protocol was followed: eligibility confirmation, informed consent, administration of the questionnaire in the local language using uniform prompts, use of neutral probing without leading participants, immediate recording of responses, and on-site completeness checks prior to closing the interview. Confidentiality was maintained throughout.

### 2.7 Statistical analysis

Statistical analysis was done using IBM SPSS Statistics V22.0. Non-parametric tests were performed as the data were not normally distributed. A Mann-Whitney *U* test was used to compare the LOC scores between males and females. Spearman correlation was performed to determine the correlation between LOC scores, age, and Socioeconomic status (SES). A multivariable analysis was conducted to assess the associations between LOC scores and sociodemographic factors. LOC was considered the dependent variable (internal vs. external) to identify sociodemographic factors associated with a predominantly internal control orientation in this population. Predictors were entered simultaneously using the Enter method based on a priori relevance: age, gender and socioeconomic status. Questionnaires were checked for completeness during data collection, and no missing data were observed for variables included in the regression model; therefore, no imputation was required. Multicollinearity among independent variables was assessed using the variance inflation factor (VIF), with a VIF >5 indicating collinearity. The Hosmer–Lemeshow test was performed to evaluate the goodness-of-fit of the final logistic regression model. Odds ratios (OR) with 95% confidence intervals (CIs) were reported (p < 0.05).

## 3 Results

**Demographic characteristics and the awareness and perception of Essential Oral Health Care (EOHC)** of the 150 study participants are shown in Table 1. Mean age was 42.82 ± 15.05 years; 52.7% were male and 49.3% were upper-lower SES. Most defined oral health as disease prevention (76.7%); 68.0% linked oral to general health (28.0% unsure). Pain relief alone was most often viewed as essential care (42.0%), and 94.7% wanted more oral health information.

**Table 1:** Demographic characteristics, Awareness and Perception of Essential Oral Health Care (EOHC) of the study participants.

Demographic data		Study group N = 150 (N, %)
Age (years)		
18-34		52 (34.7)
35-44		26 (17.3)
45-64		63 (42.0)
65-74		09 (6.0)
Total		150 (100)
Mean ± S. D		42.82 ± 15.05
Gender	Males	79 (52.7)
	Females	71 (47.3)
Socioeconomic status (SES)	Upper (I)	03 (2.0)
	Upper middle (II)	04 (2.7)
	Lower middle (III)	51 (34.0)
	Upper lower (IV)	74 (49.3)
	Lower (V)	18 (12.0)
**Awareness and Perception Questions**		
**1. Which services should be included in essential oral health care?**		
Pain relief		63 (42.0)
Tooth Extraction		02 (1.3)
Pain relief, Tooth Extraction		07 (4.7)
Pain relief, Tooth Extraction, Fillings		19 (12.7)
Pain relief, Tooth Extraction, Fillings, Gum treatment, Oral health education		22 (14.7)
Pain relief, Fillings, Gum treatment, Oral health education		10 (6.7)
Pain relief, Gum treatment, Oral health education, Regular dental check-ups		09 (6.0)
Pain relief, Tooth Extraction, Fillings, Gum treatment, Regular dental check-ups		08 (5.3)
Pain relief, Tooth Extraction, Fillings, Gum treatment, Oral health education		08 (5.3)
All of the above		02 (1.3)

The **oral health access and utilization** and **self-perceived oral health status** is shown in Table 2.

**Table 2:** Oral health access and utilization, self-perceived oral health status.

Oral Health Access and Utilization Questions	Study group N = 150 (N, %)
**1. Are oral health services easily accessible in your area?**	
Yes	62 (41.3)
No	55 36.7)
**2.Where did you seek dental care?**	**n=51 (n, %)**
Government clinic	32 (62.8)
Private clinic	14 (27.4)
Mobile camp	05 (9.8)
**3.Why did you seek dental care?**	**n=51 (n, %)**
Pain	37 (72.5)
Tooth decay	06 (11.8)
General check up	05 (9.8)
Others	03 (5.9)
**4. Are you aware of any free or subsidized oral health services in your area?**	
Yes	96 (64.0)
No	54 (36.0)
**Self-Perceived Oral Health Status Questions**	
**1. Are you currently experiencing any of the following oral health issues?**	
Toothache	58 (38.7)
Bleeding gums	34 (22.7)
Bad breath	16 (10.7)
Sensitivity	07 (4.7)
Bleeding gums and Bad breath	14 (9.3)
Toothache and Sensitivity	04 (2.7)
None	17 (11.3)
**2. How would you rate your oral health?**	
Excellent	04 (2.7)
Good	16 (10.7)
Fair	66 (44.0)
Poor	64 (42.7)
**3. Do oral problems affect your ability to eat, speak, or feel confident in daily life?**	
Yes	52 (34.7)
No	98 (65.3)

Only 34.0% visited a dentist in the past year (mainly for pain, 72.5%); government facilities were most used (62.8%). Although 41.3% perceived services as accessible, only 14.0% could afford treatment; 64.0% knew about free/subsidized care, and 18.0% had received oral health education (mostly via camps). Toothache (38.7%) and bleeding gums (22.7%) were common; 44.0% rated oral health fair and 42.7% poor.

**Barriers to accessing oral health care and experiences related to public dental facilities (PHC/CHC/Government hospital)** is shown in Table 3 (**quotes: Appendi**x-Supplementary Table 1): key barriers were cost (37.5%) and waiting time (22.5%); 69.3% reported respectful care and 87.3% felt reduced waiting and better staffing would improve use.

**Table 3:** Barriers to Accessing Oral Health Care, Barriers Related to Public Dental Facilities (PHC/CHC/Government hospital).

Barriers to Accessing Oral Health Care Questions	Study group N = 150 (N, %)
**1. Reasons for delaying or avoiding dental visits**	**n=120 (n, %)**
High cost	45 (37.5)
Long waiting time	27 (22.5)
Lack of time	04 (3.3)
Distance	13 (10.8)
Fear	11 (9.2)
High cost, Long waiting time	05 (4.1)
High cost, Fear	09 (7.6)
Long waiting time, Distance	06 (5.0)
**2. Do you feel that oral health services are welcoming and respectful in your area?**	
Yes	104 (69.3)
No	28 (18.7)
Not sure	18 (12.0)
**3. Would you be more likely to use dental services if they were available at:**	
Lower cost	84 (56.0)
Nearby	36 (24.0)
Mobile vans	07 (4.7)
Camps	10 (6.7)
After hours	08 (5.3)
Better awareness	05 (3.3)
**Barriers Related to Public Dental Facilities (PHC/CHC/Government hospital) Questions**	
**1. Have you ever visited a PHC/CHC/Government hospital for dental care?**	
Yes	104 (69.3)
No	28 18.7)
**2. If yes, Experience Category**	n = 104 (n, %)
Positive	20 (19.2)
Negative	26 (25.0)
Neutral	10 9.6)
**3. If no, reasons for not availing dental care**	n = 28 (n, %)
Long waiting times	10 (35.8)
Dental services not available	06 (21.5)
No dentist was available	04 (14.2)
Long waiting times, No water supply, No electricity	01 (3.6)
Long waiting times, Lack of dental equipment or materials	03 (10.7)
I didn't know they provided dental care	04 (14.2)

### 3.1 Locus of control and associations

LOC findings are presented in Table 4, Table 5, Table 6. Overall, 38.7% had internal LOC (≥4). Mean LOC did not differ by sex (p = 0.16). LOC correlated with SES (ρ = 0.18, p = 0.02) but not age (p = 0.93). Gender differences were significant for past-year dental visit (p = 0.0001) and delayed visits (p = 0.0008) (Table 5). In adjusted analysis (Table 6), internal LOC was associated with higher SES (upper OR 2.90; upper middle OR 2.35); age and gender were not significant. Multicollinearity was assessed using variance inflation factors (VIF) and tolerance values; no evidence of multicollinearity was observed (VIF **1.01**–**1.03**; tolerance **0.96**–**0.98**). Model fit was evaluated using the Hosmer–Lemeshow goodness-of-fit test and was acceptable (**χ^2^=5.88, p=0.66**).

**Table 4:** Locus of Control (LOC) of the study participants.

Questions	Study group N = 150 (N, %)
**1. Do you think oral health is an important part of overall health?**	
Internal LOC (Yes)	102 (68.0)
External LOC (No/Not sure)	48 (32.0)
**2. Would you be interested in knowing more about basic oral healthcare?**	
Internal LOC (Yes)	142 (94.7)
External LOC (No)	08 (5.3)
**3.Have you visited a dentist in the past year?**	
Internal LOC (Yes)	51 (34.0)
External LOC (No)	99 (66.0)
**4. Can you afford dental treatment when needed?**	
Internal LOC (Yes/Sometimes)	56 (37.3)
External LOC (No)	94 (62.7)
**5. Have you received any oral health education?**	
Internal LOC (Yes)	27 (18.0)
External LOC (No)	123 (82.0)
**6. Are you satisfied with your oral health?**	
Internal LOC (Yes)	86 (57.3)
External LOC (No)	64 (42.7)
**7.Have you ever delayed or avoided visiting a dentist?**	
Internal LOC (No)	30 (20.0)
External LOC (Yes)	120 (80.0)
**LOC Category**	
Internal LOC (Score≥4)	58 (38.7)
External LOC (Score<4)	92 (61.3)

**Table 5:** Locus of Control (LOC) of the study participants by gender.

Variables	Males Mean ± SD	Females Mean ± SD	Males Median (IQR)	Females Median (IQR)	p value
Importance of Oral health	0.72 ± 0.30	0.63 ± 0.29	1 (0–1)	1 (0–1)	0.25
Interest in learning	0.96 ± 0.10	0.93 ± 0.11	1 (1–1)	1 (1–1)	0.37
Dental visit in Past Year	0.48 ± 0.20	0.18 ± 0.08	0 (0–1)	0 (0–0)	0.0001∗
Affordability of Treatment	0.39 ± 0.18	0.35 ± 0.15	0 (0–1)	0 (0–1)	0.61
Received Oral Health Education	0.23 ± 0.09	0.13 ± 0.06	0 (0–0)	0 (0–0)	0.10
Satisfied with Oral Health	0.53 ± 0.20	0.62 ± 0.25	1 (0–1)	1 (0–1)	0.27
Delayed Dental Visit	0.30 ± 0.46	0.08 ± 0.28	0 (0–1)	0 (0–0)	0.0008∗
Mean LOC/Median LOC	3.62 ± 1.2	2.93 ± 1.34	4 (2–5)	3 (2–3)	0.16

Mann-Whitney *U* test; ∗Statistically significant at p < 0.05.

**Table 6:** Multivariate logistic regression with Locus of Control (LOC) as dependent variable.

Variables		Odds Ratio (OR)	95% CI for OR	p value
Age (per 5- year increase)		1.03	0.92-1.16	0.58
Gender (Ref: Female)		0.94	0.56-1.58	0.81
SES (Ref: Lower)	Upper (I)	2.90	1.20-7.05	0.02∗
	Upper middle (II)	2.35	1.05-5.25	0.04∗
	Lower middle (III)	1.75	0.85-3.60	0.13
	Upper Lower (IV)	1.28	0.62-2.63	0.50

CI: Confidence Interval, ∗Statistically significant.

## 4 Discussion

This study examined perceptions of Essential Oral Health Care (EOHC), barriers to public dental service use, and locus of control (LOC) in an underserved, low-resource urban community. Overall, the findings align with Indian and broader Low- and middle-income countries (LMICs) evidence showing that oral health care is frequently symptom-driven, constrained by cost and service availability, and shaped by broader social gradients. Framed within India's public health system and UHC agenda, the results underscore that strengthening EOHC requires both service readiness (workforce, supplies, infrastructure, reduced waiting times) and demand-side empowerment (oral health education and agency).^4^^,^^26^

Consistent with prior studies,^26^, ^27^, ^28^ participants commonly equated “essential” dental care with pain relief, reflecting a narrow understanding of EOHC and a care pathway oriented toward emergencies rather than prevention. This limited awareness can perpetuate delayed care-seeking and reduce demand for preventive services. In practice, the public sector remained the dominant source of care, reflecting financial constraints and reliance on government delivery in low-income settings; however, affordability remained a critical barrier, suggesting persistent gaps in financial protection for dental care.^14^^,^^29^^,^^30^

The barriers identified in this study such as cost and waiting time are repeatedly reported in previous studies,^31^^,^^32^ alongside constraints such as limited operating hours, perceived low service readiness, and referral burdens. The qualitative findings (Appendix- Supplementary Table 1) deepen this comparison by illustrating mechanisms commonly described in the public-sector literature: human resource shortages, inconsistent provider availability, restricted scope of services at PHC/CHC level, and gaps in equipment and consumables, leading to repeated referrals to higher facilities. Such readiness gaps are widely recognized as bottlenecks for integrating oral health into primary care, and they help explain why even populations that prefer or depend on government services may still delay care or seek episodic relief rather than definitive treatment.^33^^,^^34^ Self-reported oral health status and common complaints (toothache and bleeding gums) are also in line with national and regional studies^35^^,^^36^ that consistently demonstrate a high burden of untreated caries and periodontal disease in India, particularly among lower socioeconomic strata. Importantly, participants’ suggested solutions such as token/appointment systems to reduce waiting, posting additional dentists, ensuring free/subsidized care, broadening service scope, and maintaining supplies closely align with EOHC and UHC priorities for strengthening primary-level readiness and responsiveness.^6^

Integrating LOC added a psychological dimension to EOHC access. Prior health psychology and oral health studies^16^^,^^37^ suggest that individuals with greater perceived control are more likely to adopt preventive behaviours and engage with services. In this study, internal LOC was associated with higher SES, a pattern that is congruent with literature^19^^,^^24^ linking agency to care-seeking and adherence. At the same time, the qualitative accounts of long waits, provider unavailability, and repeated referrals reflect conditions that can diminish perceived control, supporting the interpretation that LOC in low-resource settings may be shaped by lived service experience as much as by individual disposition. Contrary to some previous research,^38^ our study found no correlation between LOC and either gender or age. This suggests that contextual and cultural factors may have a greater impact on LOC in this context than demographic traits.

Taken together, the findings are consistent with and extend current evidence by showing that EOHC gaps in underserved urban communities are driven by the interaction of (i) limited EOHC awareness and preventive orientation, (ii) financial and organizational barriers within the public system, and (iii) perceived agency (LOC) that appears responsive to socioeconomic and service conditions. This underscores that interventions focused only on health education may have limited impact unless accompanied by improvements in public dental service capacity and affordability.^6^^,^^27^^,^^31^

### 4.1 Strengths and limitations

The study has a few strengths. First, it includes mixed-methods elements, combining quantitative findings with representative participant quotes to better contextualize barriers and experiences. Second, it was conducted in an underserved and underrepresented urban low-resource community, improving the relevance of findings for equity-focused EOHC planning. Third, data collection was community-based and interviewer-administered, which likely enhanced participation and completeness across varying literacy levels. Finally, the study integrates locus of control into oral health access research, offering a novel psychological lens to understand EOHC perceptions and service use.

The study has several limitations. When interpreting findings at the community level, the potential for ecological bias and individual-level fallacy should be considered, and associations between SES, and LOC should therefore be interpreted cautiously. The authors acknowledge that purposive sampling may have introduced selection bias, potentially over-representing individuals who were more accessible during visits or more engaged with health services, and thereby limiting the representativeness and generalizability of the findings beyond this community. Further, although data were collected from individuals, recruitment from a single locality may still reflect local environmental and infrastructure influences, reducing extrapolation to other settings. Given the cross-sectional design, causal inference between LOC and SES is not possible. Recall and social desirability biases may have influenced self-reported oral health status and service utilization. Findings are context-specific to Trilokpuri, and may differ in higher socioeconomic urban areas or rural settings. Finally, the relatively short study period may not capture seasonal or temporal variations in service use.

### 4.2 Public health implications

Strengthening EOHC within India's public health system will require improving readiness at PHC/CHC levels through workforce augmentation, functional equipment and supplies, reduced waiting times via better patient flow, and stronger financial protection for basic oral health services. Concurrent community-based oral health education that builds self-efficacy and reinforces preventive norms may increase agency and shift utilization toward preventive care, but sustained change will depend on services being consistently available, affordable, and responsive.

## 5 Conclusion

This study highlights important gaps in awareness, perception, and utilization of Essential Oral Health Care (EOHC) services among adults in an urban low-resource community in Delhi. While most participants recognized the importance of oral health, few viewed it comprehensively, and service utilization was primarily driven by pain rather than prevention. Cost, long waiting times, and systemic deficiencies in public facilities emerged as major barriers to care.

Importantly, individuals with higher socioeconomic status were more likely to exhibit an internal Locus of Control (LOC), suggesting that empowering communities through education can strengthen self-agency in maintaining oral health. Strengthening public dental services, reducing waiting times, expanding service scope, and promoting preventive education are critical to enhance EOHC delivery and utilization.

## Participant's consent

Written informed consent was obtained from the study participants.

## Ethical clearance

Ethics approval has been obtained from the Institutional Ethics Committee, AIIMS New Delhi with letter number AIIMSA4054 dated 10.06.2025 (Ref. No.: AIIMSA4054/10.06.2025).

## Declaration of generative AI and AI-assisted technologies in the writing process

During the preparation of this work the authors used ChatGPT in order to refine the content. After using this tool/service, the authors reviewed and edited the content as needed and take full responsibility for the content of the publication.

## Funding

This research did not receive any specific grant from funding agencies in the public, commercial, or not-for-profit sectors.

## Declaration of competing interest

The authors declare that they have no known competing financial interests or personal relationships that could have appeared to influence the work reported in this paper.

## Data Availability

The datasets used and/or analyzed during the current study available from the corresponding author on reasonable request.
