# Supplementary material for: Perceptions of essential oral health care and locus of control among adults in a developing country
Source: J Oral Biol Craniofac Res. 2026 Jul 8;16(4):101487. doi: 10.1016/j.jobcr.2026.101487 (PMC13356610; doi:10.1016/j.jobcr.2026.101487)
Supplement: Multimedia component 1 [file mmc1.docx]

1. **QUESTIONNAIRE**

**Essential Oral Health Care Assessment (EOHC) and Locus of Control (LOC)Tool**

1. **Demographic Details**
2. Age: _______ years
3. Gender: ☐ Male ☐ Female ☐ Other
4. Educational level: ☐ No formal education ☐ Primary ☐ Secondary

☐ Higher Secondary ☐ Graduate and above

1. Occupation: ____________________
2. Residential area: ☐ Urban ☐ Peri urban ☐ Rural ☐
3. **Awareness and Perception of Essential Oral Health Care (EOHC)**
4. In your own words, what does “oral health” mean to you?
   __________________________
5. Which of the following services do you think should be included in basic or essential oral health care?
   ☐ Pain relief
   ☐ Tooth extraction
   ☐ Filling of cavities
   ☐ Gum treatment
   ☐ Oral health education
   ☐ Regular dental check-ups
   ☐ Others (please specify): _______________
6. **Oral Health Access and Utilization**
7. Are oral health services easily accessible in your area?
   ☐ Yes ☐ No ☐ Don’t know
8. Where do you usually seek dental care?
   ☐ Government clinic ☐ Private clinic ☐ Mobile dental camp ☐ Not applicable
9. Why did you seek dental care? : ☐ Pain ☐ Check-up ☐ Cleaning ☐ Others: ____________
10. Are you aware of any free or subsidized oral health services in your area?
    ☐ Yes ☐ No
11. **Self-Perceived Oral Health Status**
12. Do you currently have any of the following?
    ☐ Toothache ☐ Bleeding gums ☐ Loose teeth ☐ Bad breath ☐ Sensitivity
    ☐ None of the above
13. How would you rate your oral health?
    ☐ Excellent ☐ Good ☐ Fair ☐ Poor
14. Do oral problems affect your daily life (eating, speaking, confidence)?
    ☐ Yes ☐ No
15. **Barriers to Accessing Oral Health Care**

1. Do you feel that oral health services are welcoming and respectful in your area?
☐ Yes  ☐ No  ☐ Not sure

2. Reasons for delaying or avoiding dental visits? (You may choose more than one)
☐ High cost of treatment
☐ Lack of time
☐ Dental clinic is too far
☐ Fear or anxiety about dental treatment
☐ Don’t know where to go
☐ Long waiting time
☐ Belief that dental problems are not serious
☐ Cultural or traditional beliefs
☐ Language or communication problems
☐ Previous bad experience
☐ Other (please specify): _______________________

3.What is the most important reason you do not visit the dentist regularly?
☐ Cost
☐ No perceived need
☐ Lack of nearby services
☐ Fear
☐ Other: ___________________

4.Would you be more likely to use dental services if they were available at:
☐ Lower cost
☐ Nearby location
☐ Through mobile dental vans
☐ During community health camps
☐ After work hours/weekends
☐ With better awareness and information
☐ Others (please specify): ____________________

1. **Barriers Related to Public Dental Facilities (PHC/CHC/** **Government Hospitals)**

1.Have you ever visited a Primary Health Centre (PHC) or Community Health Centre (CHC) or any other centres (Government Hospitals) for dental care?
☐ Yes  ☐ No
If yes, please share your experience: _________________________________

2.If you did not visit a PHC/CHC for dental care, what were the reasons? (Tick all that apply)
☐ Dental services not available
☐ No dentist was available
☐ Poor condition of the building or dental chair
☐ No water supply
☐ No electricity
☐ Lack of dental equipment or materials
☐ Long waiting times
☐ Staff were unhelpful or rude
☐ No privacy during treatment
☐ I didn’t know they provided dental care
☐ Others (please specify): ______________________________

3.In your opinion, what problems do public dental facilities face in your area?

4.What improvements would you suggest for better dental services at PHC/CHC?

5.Do you think more people would use PHC/CHC dental services if these improvements were made?
☐ Yes  ☐ No  ☐ Not sure

**G) Locus of Control (LOC)**

1. Do you think oral health is an important part of overall health?
   ☐ Yes  ☐ No  ☐ Not sure
2. Would you be interested in knowing more about basic oral healthcare?
   ☐ Yes ☐ No
3. Have you visited a dentist in the past year? ☐ Yes ☐ No
4. Can you afford dental treatment when needed?
   ☐ Yes ☐ No ☐ Sometimes
5. Have you received any oral health education?
   ☐ Yes ☐ No
6. Are you satisfied with your oral health? ☐ Yes ☐ No
7. Have you ever delayed or avoided visiting a dentist?
   ☐ Yes  ☐ No
8. **SUPPLEMENTARY TABLES**

**Table 1. Distribution of representative quotes for – ‘Experiences with Public Dental Facilities (PHC/CHC/Government Hospital)’, ‘Barriers Identified at Public Dental Facilities’ and ‘Suggested Solutions for Improving Public Dental Facilities’**

| **Experience Type** | **% (n=104)** | **Example Quotes** |
| --- | --- | --- |
| **Positive** | 19.2% | - “Received prompt treatment.”- “I went with my child for a tooth extraction. The staff was supportive and gave us advice on oral hygiene too.”- “I visited for gum pain. The doctor cleaned my teeth, and it was free of cost. I’m satisfied with the treatment.”- “I went for a dental check-up during a health camp. The dentist was friendly and explained brushing techniques. It was a good experience.”- “During a school screening camp, I was referred to a PHC. I went and got my cavities filled.” |
| **Negative** | 25.0% | - “The doctor was available but the instruments looked old and not well maintained. I was referred to the district hospital.”- “I had to wait for 3 hours as there was only one dentist. The treatment was okay but very delayed.”- “When I went, the dentist was not available that day. I was told to come another time.”- “I had gone to a PHC for a dental filling. The doctor was helpful, but they did not have proper materials, so I had to travel to another hospital.” |
| **Neutral** | 9.6% | - “They provided pain relief for a decayed tooth but asked me to go to the dental college for further treatment.” |
| **Barrier Domain** | **%** | **Example Quotes** |
| Long Waiting Times & Overcrowding | 43.3% | - “Large number of patients causes delays.”- “Appointments are rushed or postponed.” |
| Human Resource Shortage | 22.0% | - “Only one dentist available, leading to long waits.”- “Dentist unavailable all working days.” |
| Limited Scope of Services | 14.7% | - “No facility for dentures, root canals, or braces.”- “Often referred to dental college for treatment.” |
| Infrastructure & Equipment Deficiency | 6.0% | - “Outdated and poorly maintained instruments.”- “Only one dental chair works; others are broken or unused.” |
| Consumables & Materials Shortage | 8.7% | - “No materials for fillings or root canals.”- “Lack of medicines.” |
| Accessibility & Outreach Issues | 5.3% | - “The clinic is far from my house.”- “No dental camps are held.” |
| **Suggested Solution** | **%** | **Example Quotes** |
| Reduce Waiting Time | 43.3% | - “Introduce appointment/tokens system.”- “Increase number of working days or hospital hours.” |
| Human Resource Strengthening & Free/Subsidized Treatment | 26.7% | - “Post more dentists and ensure dentists are available regularly.”- “Provide free/subsidized treatment.” |
| Broaden Scope of Services | 14.7% | - “Provide dentures and root canal treatment.”- “Braces treatment should be available.” |
| Consistent Supply of Materials & Drugs | 6.7% | - “Always keep enough stock of materials.”- “Medicines should always be available.” |
| Improve Accessibility & Outreach | 5.3% | - “Use mobile dental vans.”- “Organize regular dental camps in schools and communities.” |
| Infrastructure & Equipment Upgrade | 3.3% | - “New instruments should be used.”- “Fix dental chairs.” |
